# Prophylactic HPV Vaccination in Gynaecological Practice: Recommendations, Practices, and Challenges Reported in the ESGO-PERCH HPV Survey

**DOI:** 10.3390/vaccines14030269

**Published:** 2026-03-16

**Authors:** Joanna Kacperczyk-Bartnik, Marc Arbyn, Sophie Denoël, Esra Bilir, Nina Dhollander, Zoia Razumova, Khayal Gasimli, Andrej Cokan, Houssein El Hajj, Tibor Andrea Zwimpfer, Maria Kyrgiou, Murat Gultekin, Nicolò Bizzarri

**Affiliations:** 1 Department of Gynaecological Oncology, Maria Sklodowska-Curie National Research Institute of Oncology, 02-781 Warsaw, Poland; 2 II Department of Obstetrics, Gynaecology and Gynaecological Oncology, Medical University of Warsaw, 00-315 Warsaw, Poland; 3 Unit of Cancer Epidemiology, Belgian Cancer Centre, Sciensano, 1050 Brussels, Belgium; 4 Department of Human Structure and Repair, Faculty of Medicine and Health Sciences, University Ghent, 9000 Ghent, Belgium; 5 Department of Gynecologic Oncology, Koç University School of Medicine Department, 34450 Istanbul, Türkiye; 6 School of Medicine, American University of Sovereign Nations, Sacaton, AZ 85147, USA; 7 Department of Gynecology and Obstetrics, University Medical Center Goettingen, 37075 Goettingen, Germany; 8 Women’s and Children’s Health, Karolinska Institute, 171 77 Stockholm, Sweden; 9 Department of Gynecology and Obstetrics, J. W. Goethe University Hospital, 60590 Frankfurt, Germany; 10 Department of Gynecology and Obstetrics, Hospital zum Heiligen Geist, 60311 Frankfurt, Germany; 11 Department of Gynaecological and Breast Oncology, University Medical Centre Maribor, 2000 Maribor, Slovenia; 12 Medical Faculty Maribor, University of Maribor, 2000 Maribor, Slovenia; 13 Gynaecologic Oncology Department, Gustave Roussy, 94800 Villejuif, France; 14 Gynaecological Cancer Centre, University Hospital Basel, 4031 Basel, Switzerland; 15 Peter MacCallum Cancer Centre, Melbourne, VIC 3000, Australia; 16 Department of Biomedicine, University of Basel, 4031 Basel, Switzerland; 17 Department of Metabolism, Digestion & Reproduction-Surgery and Cancer, Faculty of Medicine, Imperial College London, London W12 0NN, UK; 18 West London Gynaecological Cancer Centre, Imperial College Healthcare NHS Trust, London W2 1NY, UK; 19 Department of Obstetrics and Gynecology, Faculty of Medicine, Hacettepe University, 06230 Ankara, Türkiye; 20 UOC Ginecologia Oncologica, Dipartimento per la Salute della Donna e Del Bambino e della Sanità Pubblica, Fondazione Policlinico Universitario a. Gemelli, IRCCS, 00168 Rome, Italy

**Keywords:** endometrial neoplasms, human papillomavirus viruses, ovarian neoplasms, primary prevention, recurrence, secondary prevention, uterine cervical dysplasia, uterine cervical neoplasms, vaccination

## Abstract

Background/Objectives: HPV vaccination is highly effective in preventing HPV-related cancers when administered before viral exposure. However, vaccination practices for patients already diagnosed with gynaecological cancers remain poorly characterized. Understanding clinicians’ perspectives and barriers is essential for optimizing preventive strategies in oncologic care. Methods: We conducted an international, web-based survey among members of the European Society of Gynaecological Oncology (ESGO) and the European Network of Young Gynaecological Oncologists (ENYGO). The questionnaire explored clinicians’ attitudes, practices, and perceived obstacles regarding HPV vaccination in patients with gynaecological cancer or pre-invasive disease across multiple clinical scenarios and age groups. Results: A total of 149 respondents from 33 countries completed the survey. Most clinicians supported HPV vaccination for patients treated for cervical precancer (78–82% for patients under 45 years), and even for invasive cervical cancer (57–62%). Recommendations varied by patients’ age, cancer type, and treatment status. For endometrial and ovarian cancer, endorsement ranged from 16% to 53%, depending on patient age. Timing of vaccination was a point of divergence: some clinicians favoured vaccination immediately after treatment for CIN2+, while others recommended delaying vaccination depending on HPV test results. Reported barriers discouraging HPV vaccination recommendations included misinformation (69.8%), lack of patient education materials (52.3%), and time constraints (48.3%), alongside economic factors and uncertainty about efficacy in oncologic settings. Conclusions: The survey shows that HPV vaccination is often recommended beyond evidence-supported indications. Randomized trials have not demonstrated a reduction in CIN2+ recurrence with adjuvant vaccination, and no evidence supports vaccination in women with invasive gynaecological cancers. These findings reveal a gap between clinical practice and available evidence, highlighting the need for clearer, evidence-based guidance.

## 1. Introduction

Human papillomavirus (HPV) infection is a well-established etiologic factor for cervical cancer and other anogenital and oropharyngeal malignancies [1]. Prophylactic HPV vaccination has demonstrated high efficacy in preventing persistent HPV infection and reducing the incidence of HPV-related precancerous lesions and cancers when administered prior to viral exposure [2,3,4,5]. Global immunization programs prioritize vaccination in early adolescence, with catch-up strategies extending into young adulthood depending on local policy and individual risk profiles [6].

However, the role of HPV vaccination in patients already diagnosed with gynaecological cancers or pre-invasive disease remains controversial. While biologic plausibility and observational data suggest potential benefits—such as reduced recurrence of cervical intraepithelial neoplasia (CIN) after excisional treatment—current evidence is limited and heterogeneous. Meta-analyses report recurrence risk reductions of up to 60% in vaccinated women compared to unvaccinated controls, yet these findings are largely derived from non-randomized studies prone to bias [7]. Randomized controlled trials, including the Dutch VACCIN trial, have not confirmed statistically significant benefits [8].

Despite these uncertainties, recommendation of HPV vaccination in this patient population remains a common clinical practice. Understanding the perspectives, practices, and barriers influencing these recommendations can be helpful for establishing clinically relevant evidence-based guidelines and for optimizing preventive strategies in oncologic care. This study aims to examine the attitudes and real-world practices of healthcare professionals regarding HPV vaccination in patients with gynaecological cancer diagnoses, focusing on timing, eligibility, and clinical considerations. Additionally, we explore perceived obstacles and potential interventions to improve integration of HPV vaccination into oncologic management.

## 2. Materials and Methods

### 2.1. Study Design and Setting

We performed a cross-sectional, international, web-based survey of members of the European Society of Gynecological Oncology (ESGO) and the European Network of Young Gynae Oncologists (ENYGO) to describe clinicians’ attitudes and everyday practices regarding prophylactic HPV vaccination across specific clinical situations and age groups. Data was collected between May and December 2024. The questionnaire comprised demographics (age, gender, training/role, years of experience), practice characteristics (setting, patient load per year, colposcopy certification), and nine sections addressing HPV vaccination knowledge, attitudes, and practices. Core outcomes were captured in Question 15 about ideal HPV vaccination recommendations and Question 16 asking about real-world practices regarding HPV vaccination recommendations across thirteen clinical scenarios and six patient age groups (≤14, 15–18, 19–24, 25–30, 31–44, ≥45 years). The full questionnaire is provided in Supplementary File S1 for transparency and reproducibility. Primary outcomes included the proportion of clinicians recommending HPV vaccination in various clinical scenarios and patient age groups (Questions 15 and 16). Secondary outcomes included preferred timing of vaccination (Questions 17–18), perceptions of barriers (Question 23), and facilitators to improve vaccination uptake (Question 24).

### 2.2. Participants and Recruitment

Eligible participants were practicing clinicians within ESGO/ENYGO (specialists/consultants in obstetrics and gynaecology or gynaecological oncology, residents/fellows in training, and other relevant professionals). Respondents were recruited via ESGO/ENYGO mailing lists, social media accounts, and onsite educational events between May and November 2024. Survey responses were collected electronically through SurveyMonkey (https://www.surveymonkey.com/, accessed on 5 December 2025). Respondents were allowed to complete the survey once. Data were stored securely and only accessible to authorized research team members. Only complete responses were included in the analysis.

Survey participants were members of ESGO/ENYGO. ESGO is the principal professional society for specialists involved in the management and research of gynaecological cancers. ENYGO is ESGO’s affiliated network for trainees and early-career clinicians, representing juniors under 40 years of age and those still in formal subspecialty training. Clinicians typically participate in ENYGO during training and transition solely to ESGO membership as they complete their specialist qualifications or surpass the age limit.

### 2.3. Statistical Analysis

All analyses were descriptive. Only complete answers were included. Categorical variables were summarized as counts and percentages; continuous variables (e.g., age) were reported as medians with interquartile ranges (IQR). For Question 15 and Question 16, the proportion of respondents recommending HPV vaccination for each clinical situation and age group was calculated. Differences between ideal recommendations and actual practice were expressed as percentage-point differences for each indication.

### 2.4. Institutional Review Board Approval

The study was conducted in accordance with the principles of the Declaration of Helsinki. The study protocol received ethical approval from the American University of Sovereign Nations School of Medicine Institutional Review Board (Institutional Review Board number IRB00009932, Assurance FWA00026409, Approval date: 30 April 2024). The study datasets are available upon request from the corresponding author.

## 3. Results

### 3.1. Participant Characteristics

A total of 149 respondents from 33 countries participated in the survey, with 96 (64.4%) practicing in Europe. The median age was 39 years (IQR: 35–47), and gender distribution was equal (49.7% female, 49.7% male). Most participants were specialists or consultants in obstetrics and gynaecology (32.9%) or gynaecological oncology (38.3%), while 14.8% were fellows in training. Clinical experience varied: 32.9% had 5–10 years post-graduation, and 24.2% had 11–15 years. Regarding cervical cancer management, 34.9% reported 5–10 years of experience. The majority worked in academic or teaching hospitals (70.5%) and 60.4% were certified for cervical dysplasia and colposcopy management. HPV vaccination of specified age groups was publicly funded for both sexes in 65.1% of respondents’ countries. Characteristics of the study group are presented in Table 1.

### 3.2. Ideal Standard of Care for HPV Vaccination Recommendations

**Figure 1:**
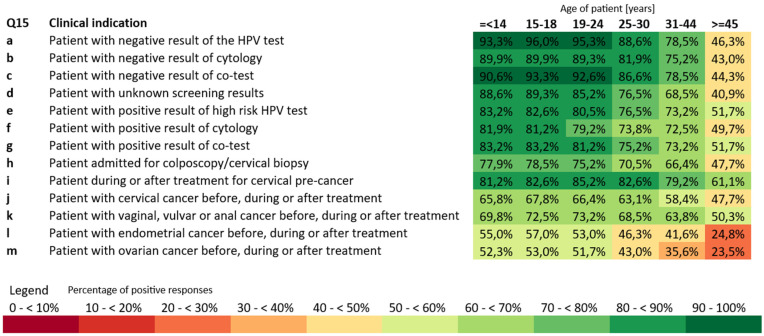
Opinions about best practice: HPV vaccination recommendations in different clinical indications and patient’s age.

Participants were asked in which clinical situations and age groups HPV vaccination should ideally be recommended to unvaccinated patients. The summary of the answers is presented in Figure 1. For patients with negative screening results—whether HPV test, cytology, or co-test—most respondents agreed that vaccination should be offered to younger age groups. Over 90% supported it for those under 18, and 95% recommended vaccination for ages 19–24. Similarly, the majority of respondents supported HPV vaccinations for women in their late twenties (around 82–88%). For older age groups the recommendation rate decreased: 78% for ages 31–44 and less than half (46%) for women of age 45 years and above. A similar trend appeared for patients with unknown screening results and for those coming in for colposcopy or treatment of cervical pre-cancer, where support ranged from about 75–82% in younger adults to just over 60% in the oldest group.

### 3.3. Real World Experience of HPV Vaccination Recommendations

**Figure 2:**
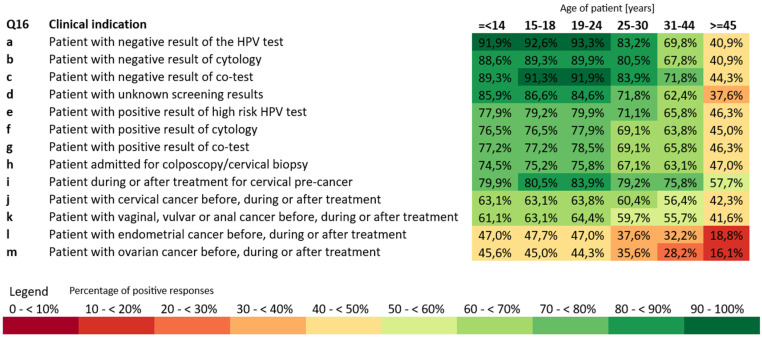
Clinical indications and age groups where gynaecologists prescribe HPV vaccination.

In everyday clinical practice, respondents reported recommending HPV vaccination less frequently than their ideal standard of care, particularly for older patients and non-cervical indications (Figure 2). For patients with negative screening results (HPV test, cytology, or co-test), the incidence of HPV vaccination recommendation remained high for younger age groups, exceeding 90% for those aged ≤18 years and 93% for ages 19–24. Support for HPV vaccination declined with age, reaching 83.2% for ages 25–30, 69.8–71.8% for ages 31–44, and 40–44% for patients ≥45 years. Similar patterns were observed for patients with unknown screening results and those undergoing colposcopy or treatment for cervical pre-cancer, where recommendations ranged from 74–80% in younger adults to 57.7% in the oldest group. For patients with positive screening results, the practice of recommending HPV vaccination was less frequent but still remained high in the context of existing evidence, with endorsement equal to 63–79% for younger age groups and 42–47% for those ≥45 years. The lowest rates were reported for non-cervical gynaecological cancers, including endometrial and ovarian cancer, where recommendations dropped below 20% for the oldest age group.

### 3.4. Discrepancies Between Ideal and Actual Recommendations

**Figure 3:**
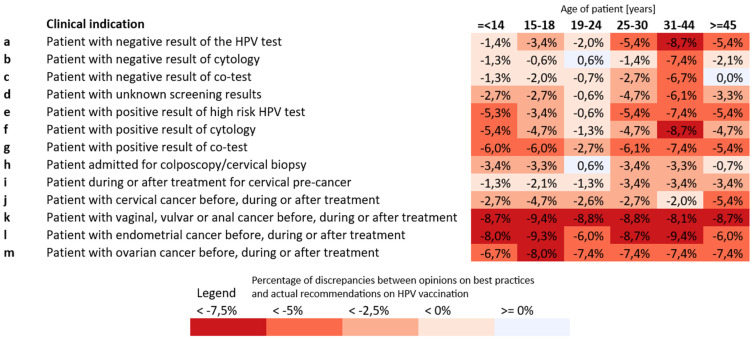
Discrepancies between ideal and real-world experience standard of care: HPV vaccination recommendations in different clinical indications.

A slight yet consistent gap was observed between what respondents considered appropriate and what they reported practicing in routine care (Figure 3). While endorsement for HPV vaccination remained high for adolescents and young adults in both ideal and real-world experience practice, actual recommendations were reported as lower across nearly all clinical situations. Among patients with negative screening results, support for vaccination declined from 93–96% (ideal) to 91–93% (actual) in younger age groups, and from 46.3% to 40.9% for those aged ≥45 years. Similar reductions were noted for patients undergoing colposcopy or treatment for cervical pre-cancer, where HPV vaccination recommendation decreased from 81–85% (ideal) to 79–83% (actual). The largest discrepancies occurred in indications beyond cervical disease, such as endometrial or ovarian cancer, where recommendations dropped by 6–9 percentage points, reaching less than 20% for older patients.

### 3.5. Timing of HPV Vaccination

Based on the survey responses, clinicians show a strong preference for administering HPV vaccination to patients with cervical intraepithelial neoplasia during admission for treatment, with 68.5% (*n* = 76) selecting this option as the optimal timing. Far fewer respondents favoured postponing vaccination to three months after treatment, whether limited to HPV-negative patients (4.5%, *n* = 5) or regardless of HPV status (8.1%, *n* = 9). Even fewer recommended initiating vaccination at six or twelve months post-treatment. Notably, only 9.9% (*n* = 11) indicated they would not recommend HPV vaccination for this patient group, and 5.4% (*n* = 6) selected other approaches.

### 3.6. Reported Barriers to Recommend HPV Vaccination to Patients

Respondents identified several obstacles that limit the integration of HPV vaccination into routine oncological care. The most frequently reported barriers were misinformation and societal beliefs about HPV infection and vaccination (69.8%), followed by lack of educational materials for patients (52.3%) and time constraints during consultations (48.3%). Communication challenges were also noted by 34.2% of participating gynaecologists. In addition, open-text responses highlighted economic factors such as vaccine cost and lack of reimbursement, uncertainty regarding efficacy in oncological settings, and insufficient evidence supporting vaccination after cancer diagnosis. Some respondents emphasized the absence of HPV vaccination in national programs and the perception that oncologic care is not the appropriate setting for preventive interventions (Table 2).

Participants identified several strategies to encourage more widespread HPV vaccination recommendations during oncological care. The most frequently suggested solutions included preparation of educational materials for patients (83.2%) and for healthcare providers (65.8%), as well as displaying educational posters in waiting areas (63.1%). Organizational interventions were also highlighted, such as workshops for healthcare providers (53.0%) and patients (47.0%). Additional suggestions provided in free-text responses emphasized improving vaccine accessibility and affordability, integrating educational prompts into electronic medical record systems, and implementing national vaccination programs to ensure HPV vaccination coverage.

## 4. Discussion

In this international survey of ESGO and ENYGO members, recommendation of HPV vaccination for patients with cervical pre-cancer was high across age groups both as an ideal management (Question 15: ~81–85% for ≤30 years; ~61% for ≥45 years) and in everyday practice (Question 16: ~79–84% for ≤30 years; ~58% for ≥45 years). These proportions indicate that many clinicians routinely recommend vaccination around the management of pre-invasive cervical disease, despite the absence of randomized evidence demonstrating reduced progression to invasive cancer or reduced development of further pre-invasive lesions after such vaccination. Our findings show that clinicians often extend HPV vaccination recommendations well beyond evidence-supported indications. This pattern of over-recommendation is particularly visible in adult women and in oncologic scenarios where no clinical benefit has been demonstrated. The tendency of gynaecologists to ‘exaggerate’ the breadth of HPV vaccine use likely reflects a combination of clinical enthusiasm, perceived patient expectations, and the lack of uniformly implemented European guidance. Importantly, this behaviour contrasts with current ESGO Prevention Committee statements, which emphasise that no recommendation can be made for routine HPV vaccination in patients with HPV-related gynaecological cancers and that evidence for adjuvant benefit after treatment of CIN2+ remains inconclusive. In this context, our survey highlights a pattern of what we describe as ‘over-indication’ of HPV vaccination—meaning that clinicians report recommending the vaccine in situations where supporting evidence is limited or currently absent. Although we recognize that this is a strong term, we chose to retain it because it captures an important and emerging practice trend revealed by our data. This refers to clinician intentions and professional judgement, not to documented patient outcomes.

HPV vaccines are most effective as primary prevention when administered before HPV infection occurs, and global guidelines prioritize adolescent vaccination for all genders, with catch-up considered for young adults depending on local policy and individual risk [9,10,11,12]. Furthermore, population-level data from Finland, Sweden, and other countries demonstrate substantial decreases in HPV-related disease incidence and cervical cancer rates among vaccinated individuals, reinforcing preventive potential. Various strategies are proposed to increase HPV vaccination rates and achieve the World Health Organization (WHO) Global Strategy for Cervical Cancer Elimination 90-70-90 targets, which aim for 90% of girls fully vaccinated with HPV vaccine by age 15, 70% of women screened with a high-performance test, and 90% of women with cervical disease receiving appropriate treatment [13,14]. These targets highlight the central role of early HPV vaccination as a proven, evidence-based intervention for cervical cancer prevention. However, this proven benefit in HPV-naïve populations does not translate into evidence for benefit as adjuvant management after treatment of pre-invasive cervical disease. The ESGO Prevention Committee explicitly states that no evidence supports HPV vaccination in patients with HPV-related gynaecological cancers and emphasizes that recommendations beyond established age-based indications should be approached with caution until randomized data are available [15].

Certain reviews and large observational cohorts suggested that adjuvant vaccination after excisional procedures may reduce CIN2+ recurrence [7,16,17]. Additionally, the risk of HPV-related disease in patients with a history of cervical precancer treatment may remain increased for as long as twenty years post-treatment [18]. However, it has been recognized that the reviews suggesting a protective effect of adding HPV vaccination to large loop excision of the transformation zone (LLETZ) were based on low quality data [19]. Some studies report even greater benefits in margin-positive cases, with recurrence risk reductions exceeding 70% [20]. These findings, combined with the theoretical advantage of limiting viral spread in the post-operative inflammatory microenvironment, support the integration of HPV vaccination into management strategies for women treated for CIN. However, these findings are often based on heterogeneous inclusion criteria and non-randomized studies, posing the risk of selection bias.

Despite expectations among healthcare professionals, the role of HPV vaccination as an adjuvant intervention after treatment of pre-invasive cervical disease remains controversial. Randomized controlled trials, such as the Dutch VACCIN trial, have not demonstrated statistically significant reductions in CIN2–3 recurrence following peri-LLETZ vaccination (RR 0.67; 95% CI 0.40–1.11; *p* = 0.11) [8]. Ongoing randomized trials aim to address the current evidence gap regarding adjuvant HPV vaccination after treatment of high-grade cervical lesions. The NOVEL trial is a multicentre, phase IV randomized controlled study evaluating the efficacy of perioperative administration of the 9-valent HPV vaccine in reducing CIN2+ recurrence following LEEP [21]. Its design includes parallel arms (vaccine vs. placebo), stratification by margin status, and a primary endpoint of histologically confirmed recurrence within 24 months. The SPERANZA trial focuses on the timing of perioperative vaccination, aiming to clarify whether it influences recurrence risk reduction [22]. SPERANZA also incorporates secondary endpoints such as quality of life and cost-effectiveness, providing a broader health-economic perspective [22]. Together, these trials are expected to generate evidence that could lead to defining future guidelines on adjuvant HPV vaccination in gynaecological oncology. However, it should be noted that a number of nationwide linkage studies integrating pathology records, cancer registries, and vaccination databases have demonstrated that HPV vaccination confers robust protection against cervical precancer and cervical cancer when administered before 15 years of age [23]. These large population-based analyses consistently show a marked decline in vaccine effectiveness with increasing age at immunization, with minimal to no measurable protection observed when vaccination occurs beyond 26 years of age [23].

Current ESGO Prevention Committee guidance emphasizes that no evidence supports routine prophylactic HPV vaccination in patients with HPV-related gynaecological cancers or pre-invasive disease [15]. Moreover, barriers such as vaccine cost, lack of reimbursement, and uncertainty regarding effectiveness in oncological settings are also arguments against recommending HPV vaccination in these adult patient populations. The ESGO opinion critically states these limitations and concludes that the current literature does not support recommending routine adjuvant vaccination in pre-invasive cervical disease. In our data, the ideal (Q15) and actual (Q16) recommendations of HPV vaccination for patients with pre-invasive cervical disease both remained high, which likely reflects clinical enthusiasm rooted in biologic plausibility (high immunogenicity, potential protection against new infections) rather than definitive clinical outcome evidence [15]. From an evidence-based medicine perspective, recommendations beyond established indications require robust data demonstrating benefit—not simply biological plausibility or absence of harm. At present, the available evidence does not support routine vaccination in several of the clinical scenarios where respondents reported recommending it. For this reason, the term ‘over-indication’ helps highlight the gap between evolving scientific evidence and current clinical attitudes. It is also important to note that new European guidelines on cervical cancer prevention and screening are currently under preparation, aiming to harmonize recommendations across countries and close existing variability in clinical practice. The divergence observed in our survey—particularly the frequent endorsement of vaccination in adult and post-diagnosis contexts—highlights why such guidelines are needed. Our results suggest that clinicians may be applying the preventive rationale of HPV vaccination more broadly than evidence supports, reinforcing the need for clearer, updated, and widely disseminated European recommendations.

It is important to distinguish between regulatory approval and evidence-based recommendation. The European Medicines Agency (EMA) authorizes certain HPV vaccines for use up to age 45, including Gardasil and Gardasil 9, which are licensed for individuals aged 9–45 years in the EU [24]. This authorization defines what is legally permissible, but it does not imply proven benefit in all age groups. Indeed, available per-protocol data do not demonstrate significant protection when vaccination is given after HPV exposure, and evidence remains insufficient to support routine vaccination in older or previously exposed women. Moreover, vaccine effectiveness depends heavily on contextual factors such as sexual culture and the average age of first sexual intercourse, which influence the window of pre-exposure protection. These nuances help explain the discrepancy observed in our survey, where clinicians often extend vaccination recommendations beyond evidence-supported indications. This pattern reinforces the need for continued educational efforts—such as webinars and targeted professional training—to improve awareness of the limits of current evidence and support consistent, evidence-based counselling.

Our findings also highlight a clear need for enhanced professional communication. To ensure that evolving evidence is correctly understood and consistently applied, more webinars, educational initiatives, and structured dissemination efforts will be essential. These activities may help align daily clinical practice with the best available data and reduce uncertainty regarding HPV vaccination in adult or previously exposed women. Multiple articles available in the literature prove that adequate training and education of clinicians increases the HPV vaccination coverage in dedicated populations aligning with evidence-based recommendations [25,26,27].

Because our survey was distributed through ESGO and ENYGO channels, we were not able to calculate a formal response rate, and we acknowledge that this type of voluntary participation always brings a risk of selection bias. At the same time, the fact that 149 clinicians from 33 different countries chose to take part gives the dataset a genuinely international character that is not often achievable in specialist surveys. This broad geographical spread likely improves the overall relevance of the findings, even if the participants may represent clinicians who are more engaged or more interested in HPV prevention. We also recognize the possibility of non-response bias, as those who feel strongly about vaccination—positively or negatively—may have been more motivated to complete the questionnaire. Still, capturing the views of clinicians who are actively involved in preventive work has value, as these are often the people shaping everyday practice.

We propose to frame this diversity as a strength, as respondents represented a wide range of clinical systems, funding models and vaccination policies, which mirrors the varied realities of HPV prevention across Europe and beyond. This aligns well with the direction set by the January 2026 update of the European Code Against Cancer, which stresses the importance of consistent, evidence-based HPV vaccination strategies across countries [28]. Against this background, our results offer a useful snapshot of how clinicians currently think and practice across very different settings, while we remain transparent about the methodological limitations that come with any voluntary international survey.

## 5. Conclusions

Results of the ESGO-PERCH survey confirm that HPV vaccination is frequently recommended beyond established indications, revealing some level of over-indication of HPV vaccination based on expert opinion. Over-indication means vaccination in situations where the clinical benefit of the vaccine is not proven by evidence. While observational studies suggest potential benefits of HPV vaccination in reducing recurrence after treatment for cervical precancer, no such evidence exists for women diagnosed with invasive gynaecological cancers. Current ESGO recommendations emphasize caution. These findings highlight the discrepancy between current clinical practice and the available evidence, reinforcing the need for clearer, evidence-based recommendations in scenarios where randomized trials have shown no reduction in CIN2+ recurrence and where no data support vaccination for women with invasive gynaecological cancers.

## Figures and Tables

**Table 1:** Characteristics of participating gynaecologists.

Characteristic	Category	*n* (%), Total *n* = 149
Age [years]	Median (IQR *)	39 (35–47)
Gender	Female	74 (49.7)
	Male	74 (49.7)
	Other	1 (0.7)
Years since medical degree graduation	<5	13 (8.7)
	5–10	49 (32.9)
	11–15	36 (24.2)
	16–20	14 (9.4)
	21–25	14 (9.4)
	26–30	8 (5.4)
	>30	15 (10.1)
Experience in cervical dysplasia/cancer management [years]	<5	44 (29.5)
	5–10	52 (34.9)
	11–15	21 (14.1)
	16–20	8 (5.4)
	21–25	10 (6.7)
	26–30	5 (3.4)
	>30	7 (4.7)
	Not applicable	2 (1.3)
Professional level	Specialist/consultant in Obstetrics and Gynaecology	49 (32.9)
	Resident in Obstetrics and Gynaecology	15 (10.1)
	Specialist/consultant in Gynaecological Oncology	57 (38.3)
	Fellow in Gynaecological Oncology	22 (14.8)
	Other	6 (4.0)
Practice setting	Academic/Teaching hospital	105 (70.5)
	Comprehensive cancer centre	22 (14.8)
	Community/city hospital	24 (16.1)
	Private hospital	28 (18.8)
	Doctor’s office/outpatient care	22 (14.8)
	Other	2 (1.3)
Certification in cervical dysplasia treatment/colposcopy	Yes	90 (60.4)
ESGO-accredited training centre	Yes	39 (26.2)
Centre volume (new primary gynaecological malignancies in 2023)	<100	51 (34.2)
	100–500	73 (49.0)
	500–1000	17 (11.4)
	>1000	8 (5.4)
National HPV vaccination program status	Publicly funded for girls and boys	97 (65.1)
	Publicly funded for girls only	18 (12.1)
	Available, no public funding	31 (20.8)
	Not available	3 (2.0)

* IQR—Interquartile range.

**Table 2:** Reported barriers to HPV vaccination recommendation in gynaecological oncological care.

Barrier	Number of Respondents	Percentage (%)
Fake news and societal beliefs about HPV	104	69.8
Lack of educational materials for patients	78	52.3
Lack of time during consultations	72	48.3
Communication barriers	51	34.2
Other (cost, lack of reimbursement, uncertainty about efficacy, lack of evidence, structural issues)	22	14.8

## Data Availability

The study datasets are available upon request from the corresponding author.

## Supplementary Materials

The following supporting information can be downloaded at: https://www.mdpi.com/article/10.3390/vaccines14030269/s1, File S1: ESGO-PERCH questionnaire on prophylactic HPV vaccination recommendations in gynaecological practice.

## Abbreviations

CIN	Cervical intraepithelial neoplasia
ESGO	The European Society of Gynaecological Oncology
ENYGO	The European Network of Young Gynaecological Oncologists
HPV	Human papillomavirus
IQR	Interquartile range
LLETZ	Large loop excision of the transformation zone
PERCH Project	Partnership to contrast HPV Project https://www.projectperch.eu/
WHO	World Health Organization

## References

[1] Lycke K.D., Steben M., Garland S.M., Woo Y.L., Cruickshank M.E., Perkins R.B., Bhatla N., Ryser M.D., Gravitt P.E., Hammer A. (2025). An updated understanding of the natural history of cervical human papillomavirus infection-clinical implications. Am. J. Obstet. Gynecol..

[2] Arbyn M., Xu L. (2018). Efficacy and safety of prophylactic HPV vaccines. A Cochrane review of randomized trials. Expert Rev. Vaccines.

[3] Arbyn M., Xu L., Simoens C., Martin-Hirsch P.P. (2018). Prophylactic vaccination against human papillomaviruses to prevent cervical cancer and its precursors. Cochrane Database Syst. Rev..

[4] Schiller J., Lowy D. (2018). Explanations for the high potency of HPV prophylactic vaccines. Vaccine.

[5] Lei J., Ploner A., Elfström K.M., Wang J., Roth A., Fang F., Sundström K., Dillner J., Sparén P. (2020). HPV Vaccination and the Risk of Invasive Cervical Cancer. N. Engl. J. Med..

[6] Advisory Committee on Immunization Practices (ACIP) ACIP Recommendations: Human Papillomavirus (HPV) Vaccine 2024. https://www.cdc.gov/acip-recs/hcp/vaccine-specific/hpv.html.

[7] Jentschke M., Kampers J., Becker J., Sibbertsen P., Hillemanns P. (2020). Prophylactic HPV vaccination after conization: A systematic review and meta-analysis. Vaccine.

[8] van de Laar R.L., Hofhuis W., Duijnhoven R.G., Bekkers R.L., Smedts H.P., Nieuwenhuyzen-de Boer G.M., van Beekhuizen H.J., van de Swaluw J.A., van Baal M.W., van den Tillaart S.S. (2025). Adjuvant prophylactic human papillomavirus vaccination for prevention of recurrent high-grade cervical intraepithelial neoplasia lesions in women undergoing lesion surgical treatment (VACCIN): A multicentre, phase 4 randomised placebo-controlled trial in the Netherlands. Lancet Obstet. Gynaecol. Women’s Health.

[9] Cortés B., Ocampo R., Porras C., Liu D., Gail M.H., Sierra M.S., Herrero R., Lowy D.R., Carvajal L.J., Kemp T.J. (2025). Human papillomavirus (HPV) type 16 and type 18 antibody concentrations after a single dose of bivalent HPV vaccine in girls aged 9–14 years compared with three doses of quadrivalent HPV vaccine in women aged 18–25 years in Costa Rica (PRIMAVERA): A non-randomised, open-label, immunobridging, non-inferiority trial. Lancet Infect. Dis..

[10] Lehtinen M., Paavonen J., Wheeler C.M., Jaisamrarn U., Garland S.M., Castellsagué X., Skinner S.R., Apter D., Naud P., Salmerón J. (2012). Overall efficacy of HPV-16/18 AS04-adjuvanted vaccine against grade 3 or greater cervical intraepithelial neoplasia: 4-year end-of-study analysis of the randomised, double-blind PATRICIA trial. Lancet Oncol..

[11] Kreimer A.R., Porras C., Liu D., Hildesheim A., Carvajal L.J., Ocampo R., Romero B., Gail M.H., Cortes B., Sierra M.S. (2025). Noninferiority of One HPV Vaccine Dose to Two Doses. N. Engl. J. Med..

[12] Bilir E., Saçıntı K.G., Kacperczyk-Bartnik J., Gultekin M. (2024). FDA approves the first HPV self-collection solutions. Int. J. Gynecol. Cancer.

[13] Kacperczyk-Bartnik J., El Hajj H., Tóth I., Bizzarri N., Tóth R., Razumova Z., Zwimpfer T.A., Taumberger N., Bilir E., Strojna A. (2025). Declaration on cervical cancer elimination: Literature review and perspectives from early-career clinicians. Int. J. Gynecol. Cancer.

[14] Wilailak S., Kengsakul M., Kehoe S. (2025). Strategic approaches for global cervical cancer elimination: An update review and call for national action. Int. J. Gynaecol. Obstet..

[15] Bizzarri N., Kyrgiou M., De Vincenzo R., Zapardiel I., Razumova Z., Taumberger N., Toth I., Theofanakis C., Gultekin M., Joura E.A. (2025). Prophylactic HPV vaccination in HPV-related gynecologic cancers: European Society of Gynecological Oncology (ESGO) prevention committee opinion. Int. J. Gynaecol. Obstet..

[16] Dvořák V., Petráš M., Lomozová D., Dlouhý P., Lesná I.K., Pilka R. (2024). Reduced risk of CIN2+ recurrence in women immunized with a 9-valent HPV vaccine post-excision: Retrospective cohort study. Hum. Vaccines Immunother..

[17] Joura E., Kjaer S.K., Bautista O., Luxembourg A., Saah A., Giuliano A. (2026). Effect of Prior 9-Valent Human Papillomavirus Vaccination on Subsequent Lower Genital Tract Dysplasia After Cervical Excisional Surgery. Obstet. Gynecol..

[18] Ebisch R.M., Rutten D.W., IntHout J., Melchers W.J., Massuger L.F., Bulten J., Bekkers R.L., Siebers A.G. (2017). Long-lasting increased risk of human papillomavirus–related carcinomas and premalignancies after cervical intraepithelial neoplasia grade 3: A population-based cohort study. J. Clin. Oncol..

[19] Kechagias K.S., Kalliala I., Bowden S.J., Athanasiou A., Paraskevaidi M., Paraskevaidis E., Dillner J., Nieminen P., Strander B., Sasieni P. (2022). Role of human papillomavirus (HPV) vaccination on HPV infection and recurrence of HPV related disease after local surgical treatment: Systematic review and meta-analysis. BMJ.

[20] Petráš M., Lomozová D., Dvořák V., Malinová J., Trnková M., Fišer I., Dlouhý P., Rosina J., Lesná I.K. (2025). Early and long-term effects of prophylactic and post-excision human papillomavirus vaccination on recurrent high-grade cervical intraepithelial neoplasia relative to margin status: A retrospective cohort study in the Czech Republic. Lancet Reg. Health Eur..

[21] Kyrgiou M. (2023). Nonavalent Prophylactic HPV Vaccine (GARDASIL9) After Local Conservative the NOVEL Trial (NOVEL). https://clinicaltrials.gov/study/NCT03979014.

[22] Ghelardi A., Parazzini F., Martella F., Pieralli A., Bay P., Tonetti A., Svelato A., Bertacca G., Lombardi S., Joura E.A. (2018). SPERANZA project: HPV vaccination after treatment for CIN2. Gynecol. Oncol..

[23] Arbyn M., Rousta P., Bruni L., Schollin Ask L., Basu P. (2024). Linkage of individual-patient data confirm protection of prophylactic human papillomavirus vaccination against invasive cervical cancer. J. Natl. Cancer Inst..

[24] European Medicines Agency (2025). Gardasil 9: EPAR—Product Information. https://www.ema.europa.eu/en/documents/product-information/gardasil-9-epar-product-information_en.pdf.

[25] D’AMbrosio F., Sezzatini R., Bucciardini R., Maida A., Nisticò A., De Vito E., Ricciardi W., Boccia S., Calabrò G.E. (2025). Educational interventions and communication strategies to improve HPV immunization uptake: A systematic literature review. Front. Public Health.

[26] Gilkey M.B., Grabert B.K., Heisler-MacKinnon J., Bjork A., Boynton M.H., Kim K., Dailey S.A., Liu A., Todd K.G., Schauer S.L. (2022). Coaching and Communication Training for HPV Vaccination: A Cluster Randomized Trial. Pediatrics.

[27] Wang R., Liu L., Dodd S., Graham S., Rook S., Ericson L., Plax K., Barker A., McKay V., I Silver M. (2026). A Multicomponent Strategy to Increase Human Papillomavirus Vaccination Rates in Primary Care: A Cluster Randomized Clinical Trial. JAMA Netw. Open.

[28] Espina C., Ritchie D., Riboli E., Kromhout H., Franceschi S., Lansdorp-Vogelaar I., Marteau T.M., Bakogianni I., Vilahur N., Alberts C.J. (2026). European Code Against Cancer 5th edition: 14 ways you can help prevent cancer. Lancet Reg. Health Eur..

