# Supplementary material for: Prophylactic HPV Vaccination in Gynaecological Practice: Recommendations, Practices, and Challenges Reported in the ESGO-PERCH HPV Survey
Source: Vaccines (Basel). 2026 Mar 16;14(3):269. doi: 10.3390/vaccines14030269 (PMC13030639; doi:10.3390/vaccines14030269)
Supplement: Supplementary file 1 [file vaccines-14-00269-s001.zip › vaccines-4137528-supplementary.pdf]

Prophylactic HPV vaccination recommendations – practices among ESGO and ENYGO members

We would like to invite you to participate in a new ESGO survey.

Our aim is to assess HPV vaccination recommendations practices among clinicians managing patients with gynaecologic cancer.

The estimated completion time is 10 minutes. Your input in obtaining the results is very important and we are very grateful for your help.

Your participation in this survey is voluntary. If you choose to participate, your responses will be kept confidential. The information collected during this survey will be used for research purposes. Only the research team and authorized personnel will have access to the data. Data will be stored securely and will be retained until the publication of the anonymized data analysis in a peer-reviewed journal.
By proceeding with the survey, you indicate that you have read the information provided, understood the purpose and procedures, and voluntarily agree to participate. You may print a copy of this consent form for your records.
If you have any questions or concerns about the survey, please contact the ESGO Office https://esgo.org/contact adminoffice@esgo.org
For data protection reasons, we ask you not to include personal data in the open text fields (with the exception of your optional contact information at the end of the survey).
We appriecиate every shared answer and point of view. Thank you very much for your help in data collection on this important topic.

* 1. **Years of clinical experience after completing medical degree:**

- < 5
- 5-10
- 11-15
- 16-20
- 21-25
- 26-30
- > 30

* 2. **Years of clinical experience in the management of cervical dysplasia and cervical cancer:**

- < 5
- 5-10
- 11-15
- 16-20
- 21-25
- 26-30
- > 30
- Not applicable

* 3. **Level of education**

- Specialist/consultant in Obstetrics and Gynaecology
- Resident (in training) in Obstetrics and Gynaecology
- Specialist/consultant in Gynaecological Oncology
- Fellow (in training) in Gynaecological Oncology
- Other (please specify)

* 4. **Gender**

- Female
- Male
- Other

* 5. **Age**

* 6. **Country of practice:**

* 7. **Places of work** (please choose all eligible answers)**:**

- ☐ Academic/Teaching hospital
- ☐ Comprehensive cancer centre
- ☐ Community/city hospital
- ☐ Private hospital
- ☐ Doctor’s office/outpatient care
- ☐ Other (please specify)

* 8. **Are you certified for cervical dysplasia, colposcopy and cervical pathology management by any national or international society for gynaecology or oncology?**

- ○ Yes
- ○ No

* 9. **Do you work in an ESGO accredited centre for training?**

- ○ Yes
- ○ No

* 10. **Which of the following options describes best the current HPV vaccination status in your country?**

- ○ Available with the opportunity to finance from public resources for both boys and girls
- ○ Available with the opportunity to finance from public resources for girls
- ○ Available but without the opportunity to finance from public resources
- ○ Not available

* 11. **During the management of patient with cervical (pre-)cancer, in which steps of the process do you actively participate?** (you can select more than one answer)

- ☐ Cervical cancer screening (collection of cervical specimens)
- ☐ Diagnostic workup of precancer (colposcopy/biopsy)
- ☐ Treatment of precancer
- ☐ Follow-up after treatment of precancer
- ☐ Surgical treatment of cancer
- ☐ Other treatment of cancer
- ☐ Palliative care
- ☐ Other (please specify)

* 12. **Number of new patients with a primary gynaecological malignancy treated in your centre in 2023:**

- ◯ <100
- ◯ 100-500
- ◯ 500-1000
- ◯ >1000

* 13. **How often do you discuss cervical cancer screening when a patient consults you for uterine cervix disease?**

- ◯ Never
- ◯ Rarely
- ◯ Sometimes
- ◯ Often
- ◯ Always

* 14. **How often do you discuss cervical cancer screening when a patient consults you for other conditions than uterine cervix disease?**

- ◯ Never
- ◯ Rarely
- ◯ Sometimes
- ◯ Often
- ◯ Always

* 15. **In which clinical situation(s) and patient's age do you think it would be appropriate to recommend HPV vaccination to an unvaccinated patient? (What should be the ideal standard of care)**

You can select more than one answer

	≤14 y/o	15-18 y/o	19-24 y/o	25-30 y/o	31-44 y/o	≥ 45 y/o
Patient with negative result of the HPV test						
Patient with negative result of cytology						
Patient with negative result of co-test (negative HPV test and negative cytology)						
Patient with unknown screening results						
Patient with positive result of high risk HPV test						
Patient with positive result of cytology (ASCUS, LSIL, HSIL, ASC-H, AGC, AIS, SCC)						
Patient with positive result of co-test (positive high risk HPV test and/or positive cytology)						
Patient admitted for colposcopy/ cervical biopsy						
Patient during or after treatment for cervical pre-cancer						
Patient with cervical cancer before, during or after treatment						
Patient with vaginal, vulvar or anal cancer before, during or after treatment						
Patient with endometrial cancer before, during or after treatment						
Patient with ovarian cancer before, during or after treatment						

* 16. **In which clinical situation(s) and patient's age do you actually recommend HPV vaccination to an unvaccinated patient in your everyday practice?**

You can select more than one answer

	≤14 y/o	15-18 y/o	19-24 y/o	25-30 y/o	31-44 y/o	≥ 45 y/o
Patient with negative result of the HPV test						
Patient with negative result of cytology						
Patient with negative result of co-test (negative HPV test and negative cytology)						
Patient with unknown screening results						
Patient with positive result of high risk HPV test						
Patient with positive result of cytology (ASCUS, LSIL, HSIL, ASC-H, AGC, AIS, SCC)						
Patient with positive result of co-test (positive high risk HPV test and/or positive cytology)						
Patient admitted for colposcopy/ cervical biopsy						
Patient during or after treatment for cervical pre-cancer						
Patient with cervical cancer before, during or after treatment						
Patient with vaginal, vulvar or anal cancer before, during or after treatment						
Patient with endometrial cancer before, during or after treatment						
Patient with ovarian cancer before, during or after treatment						

* 17. **What do you think is the most appropriate timing for the first dose of HPV vaccine administration in patients diagnosed with cervical intraepithelial neoplasia (who previously did not receive HPV vaccine)?**

- ◯ During admission for treatment
- ◯ 3 months after treatment in HPV negative patients
- ◯ 3 months after treatment regardless of the HPV test result
- ◯ 6 months after treatment in HPV negative patients
- ◯ 6 months after treatment regardless of the HPV test result
- ◯ 12 months after treatment in HPV negative patients
- ◯ 12 months after treatment regardless of the HPV test result
- ◯ I would not recommend HPV vaccination in this group of patients
- ◯ Other (please specify)

* 18. **What do you think is the most appropriate timing for the first dose of HPV vaccine administration in patients diagnosed with cervical cancer (who previously did not receive HPV vaccine)?**

- ◯ During admission for treatment
- ◯ 3 months after treatment in HPV negative patients
- ◯ 3 months after treatment regardless of the HPV test result
- ◯ 6 months after treatment in HPV negative patients
- ◯ 6 months after treatment regardless of the HPV test result
- ◯ 12 months after treatment in HPV negative patients
- ◯ 12 months after treatment regardless of the HPV test result
- ◯ I would not recommend HPV vaccination in this group of patients
- ◯ Other (please specify)

* 19. **Do you recommend HPV vaccinations to women who work as healthcare professionals and previously did not receive HPV vaccine?**

- ◯ Yes
- ◯ No

* 20. **As a gynaecologist, have you already been vaccinated against HPV yourself?**

- ◯ Yes
- ◯ No

* 21. **Do you discuss with your patients about the HPV vaccination recommendations for their adolescent children?**

- ◯ Yes if they have daughter(s)
- ◯ Yes regardless of the sex of their child
- ◯ No

* 22. **Where do you seek additional knowledge regarding current HPV vaccinations recommendations?** (you can select more than one answer)

- ☐ Medical literature
- ☐ Medical congresses and conferences
- ☐ Information from industry
- ☐ Information from the public health sector
- ☐ I do not seek additional knowledge regarding current HPV vaccinations recommendations
- ☐ Other (please specify)

* 23. **What could be the potential barriers discouraging the recommendation of HPV vaccination during routine oncological care?** (Please choose up to 3 most relevant barriers)

- ☐ Lack of time
- ☐ Lack of educational materials for patients
- ☐ Communication barriers
- ☐ Fake news and beliefs about HPV infections and HPV vaccinations in the society
- ☐ Other (please specify)

* 24. **What could be the ways of encouraging more widespread HPV vaccination recommendations?** (Please choose up to 3 best solutions)

- ☐ Preparation of educational materials for healthcare providers
- ☐ Preparation of educational materials for patients
- ☐ Hanging educational posters about HPV vaccinations programmes/recommendations in the waiting room
- ☐ Organization of workshops for healthcare providers
- ☐ Organization of workshops for patients
- ☐ Organization of workshops for patients' families
- ☐ Other (please specify)

25. **Would you like to suggest further research topics about prevention of HPV related disease?**
